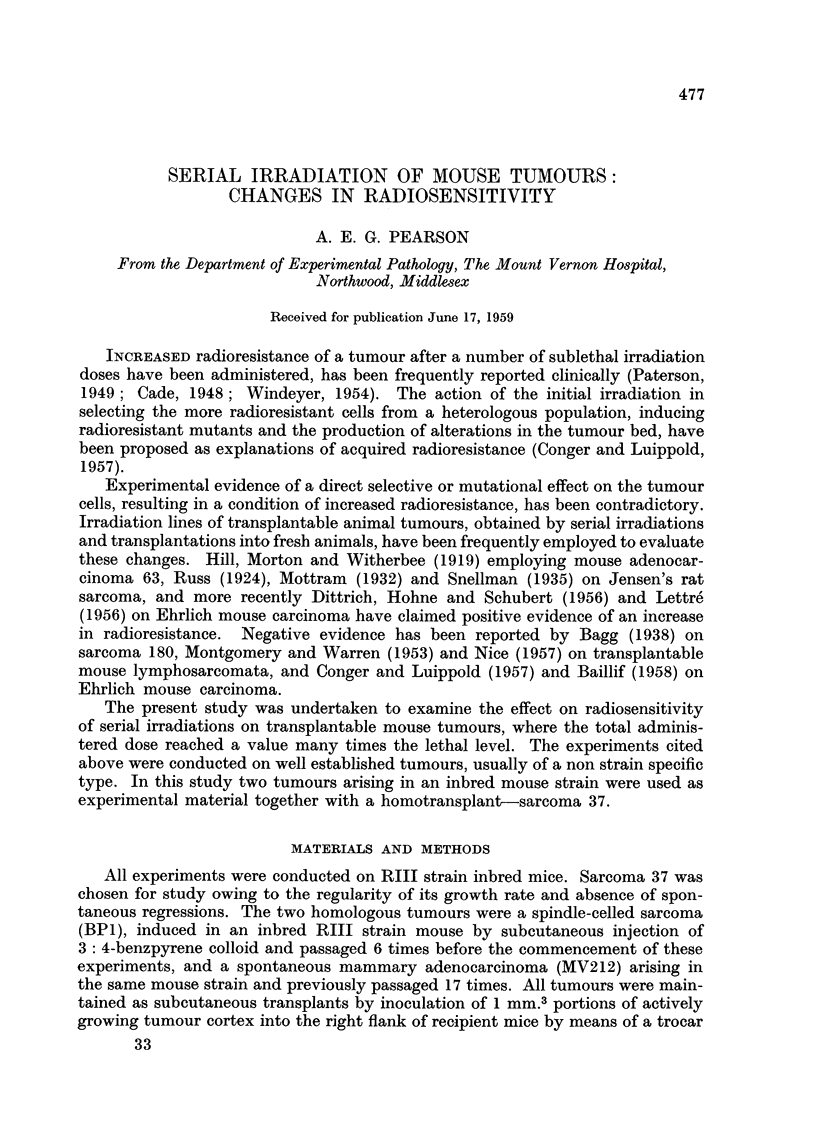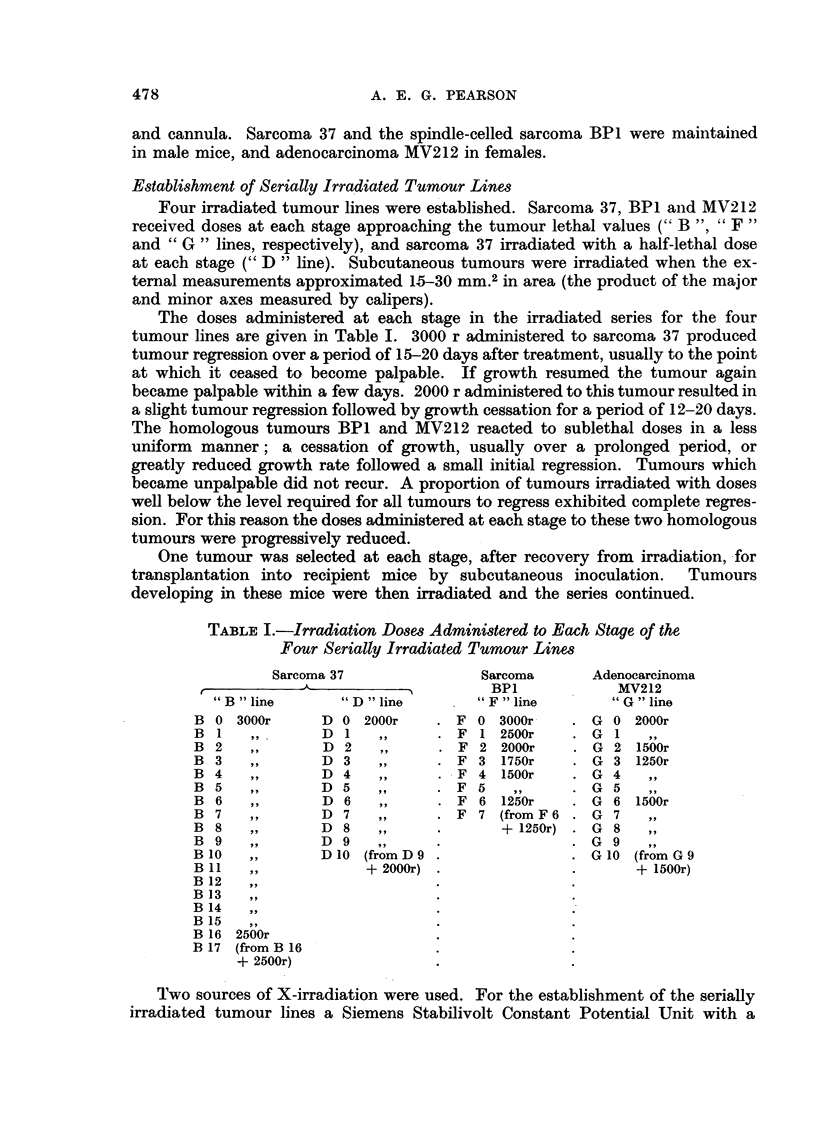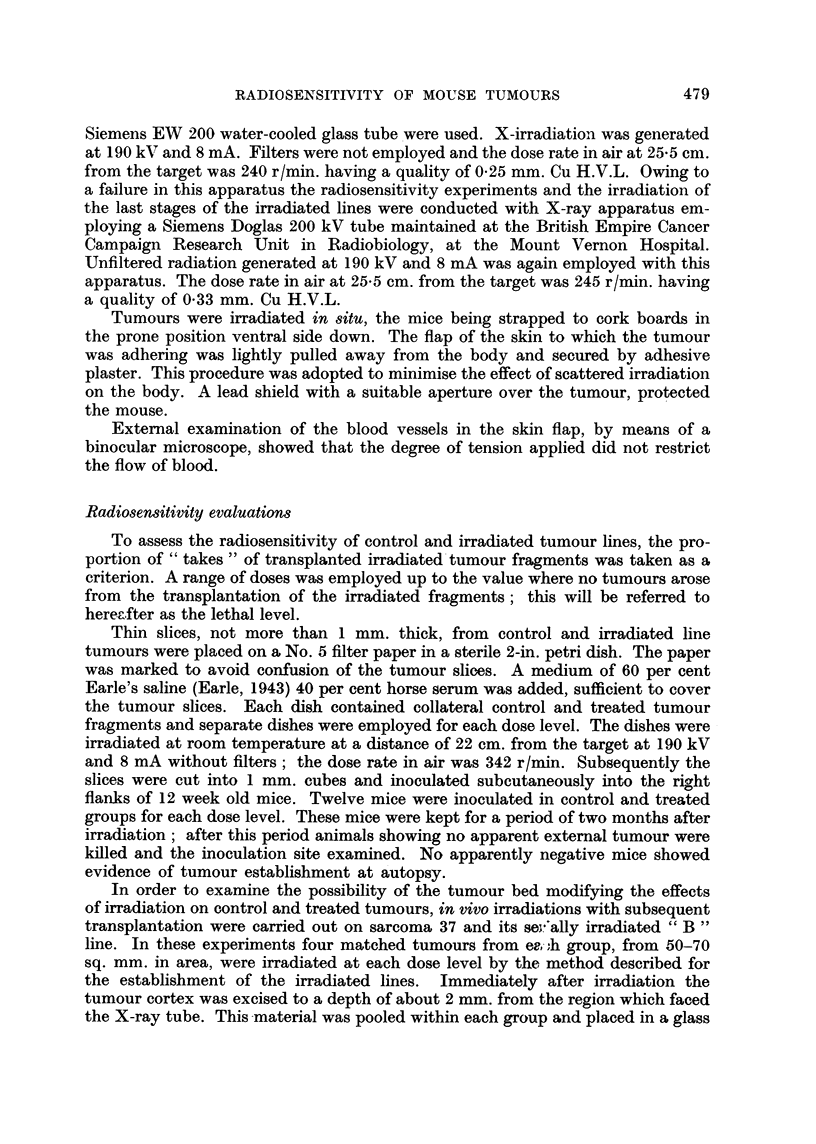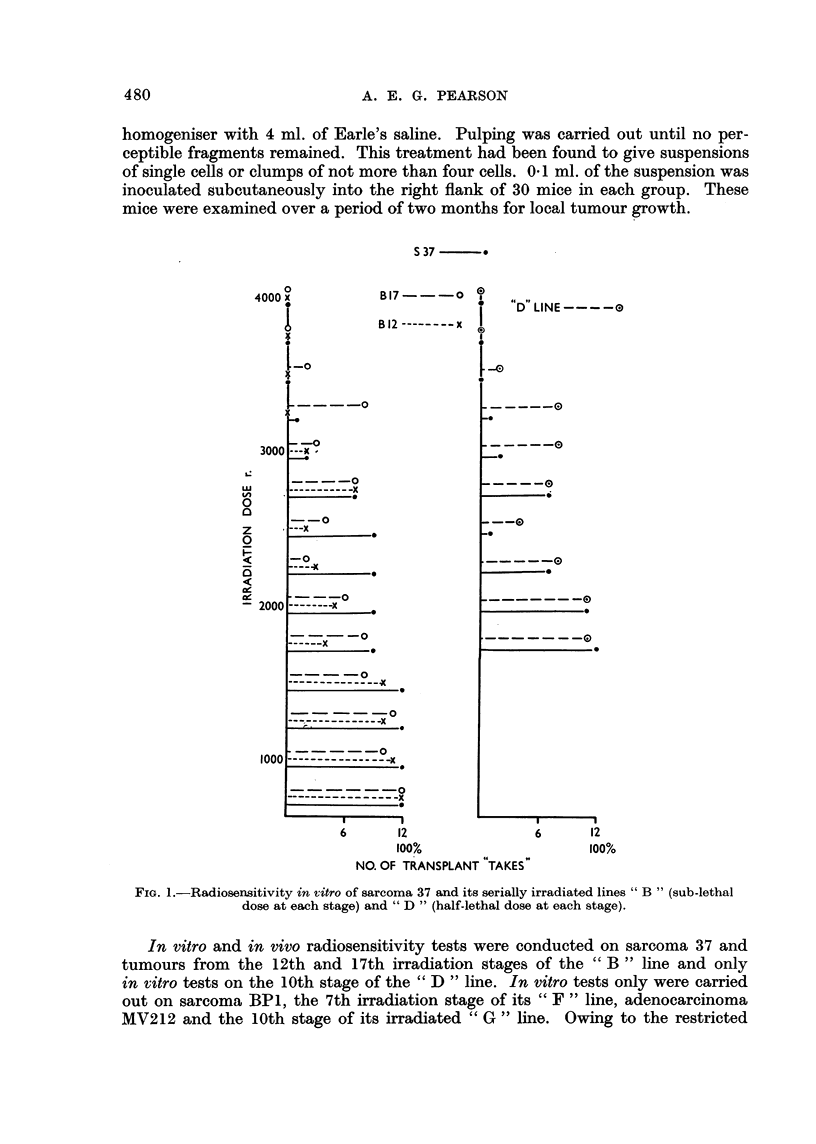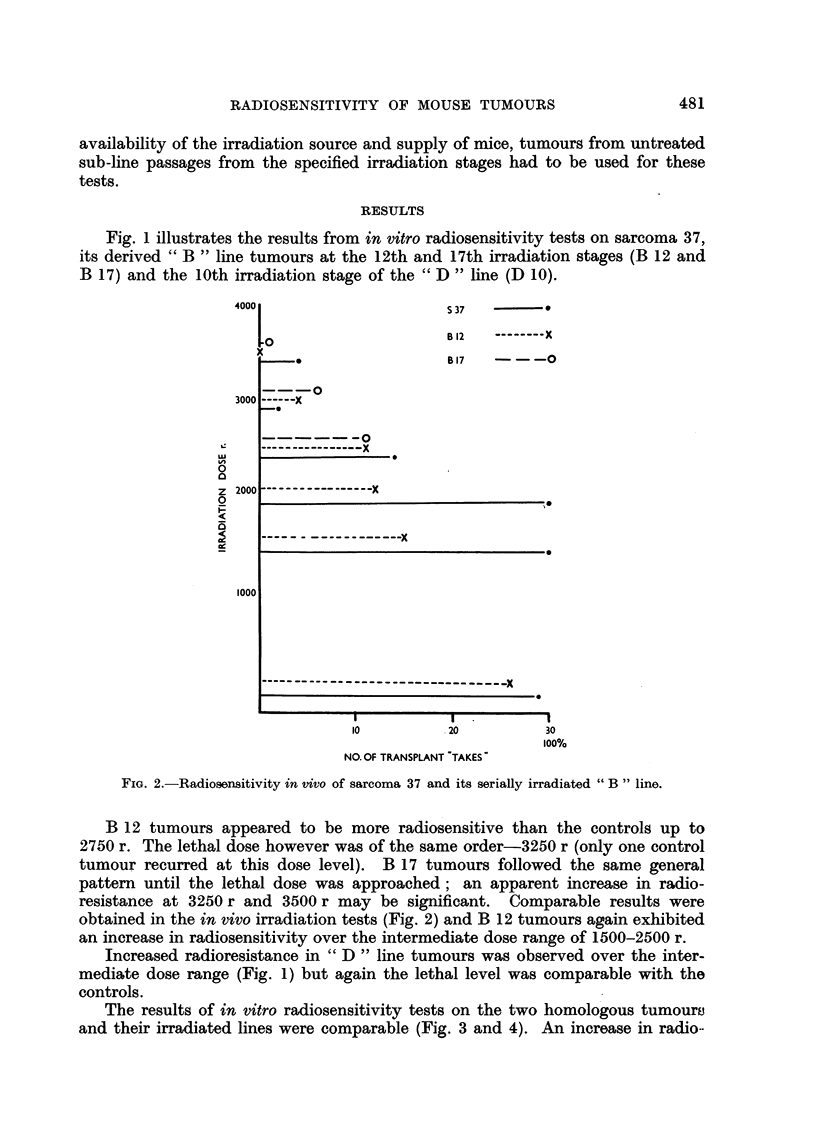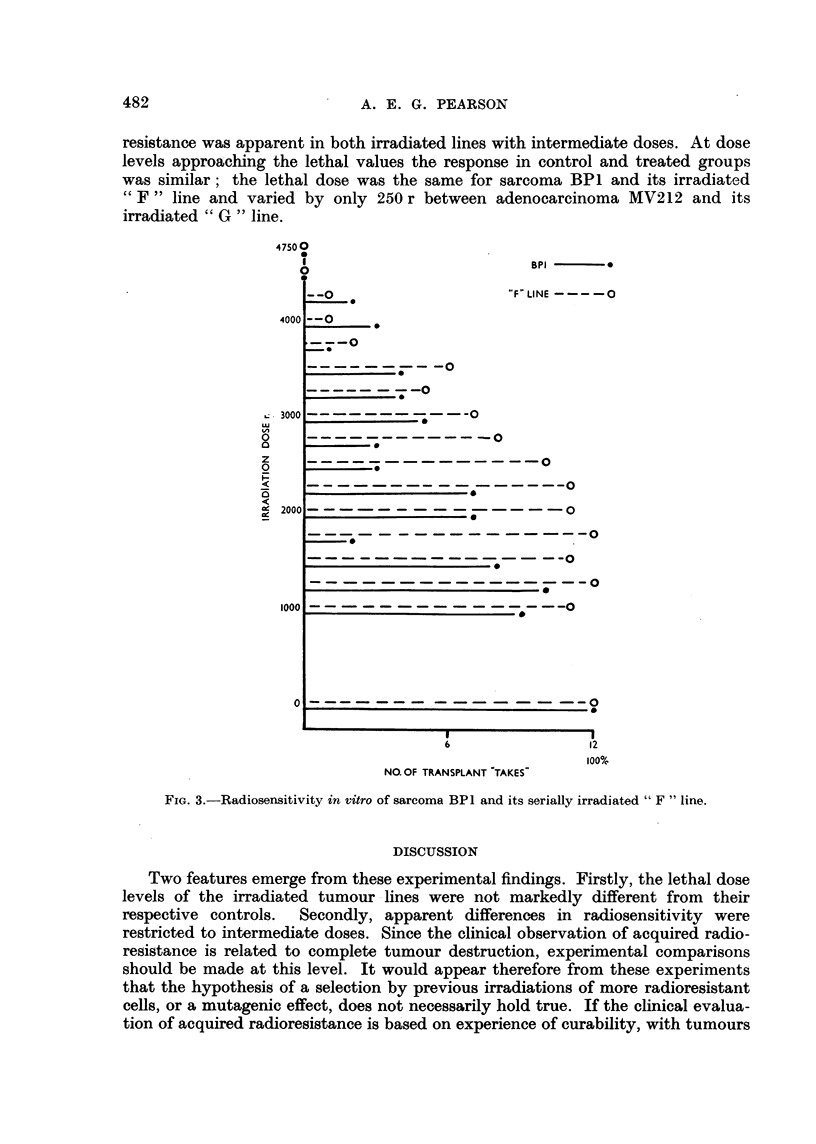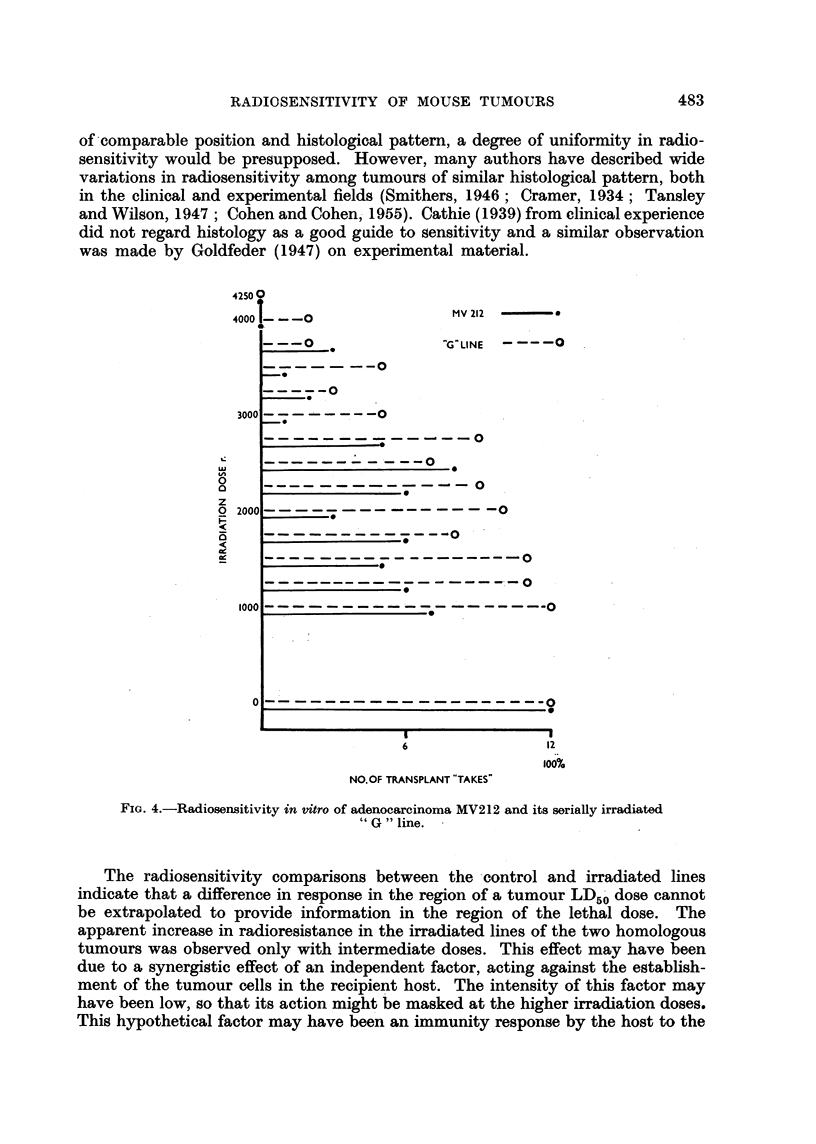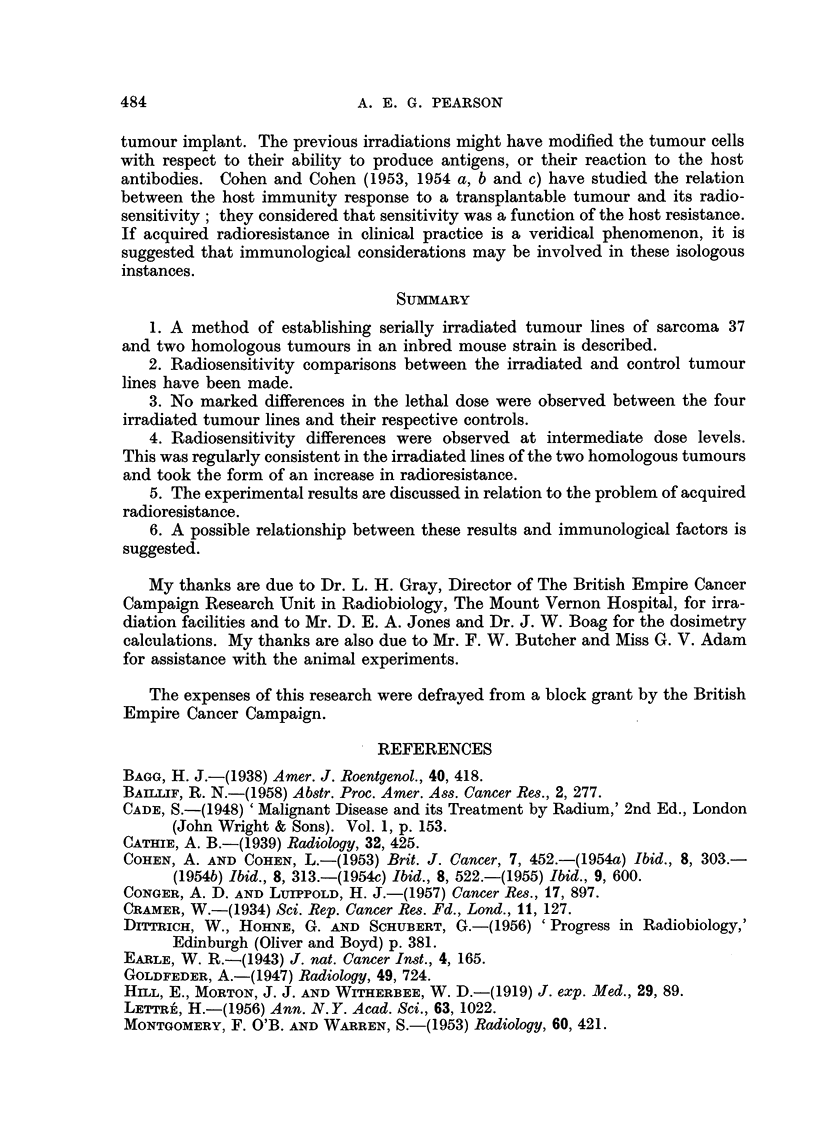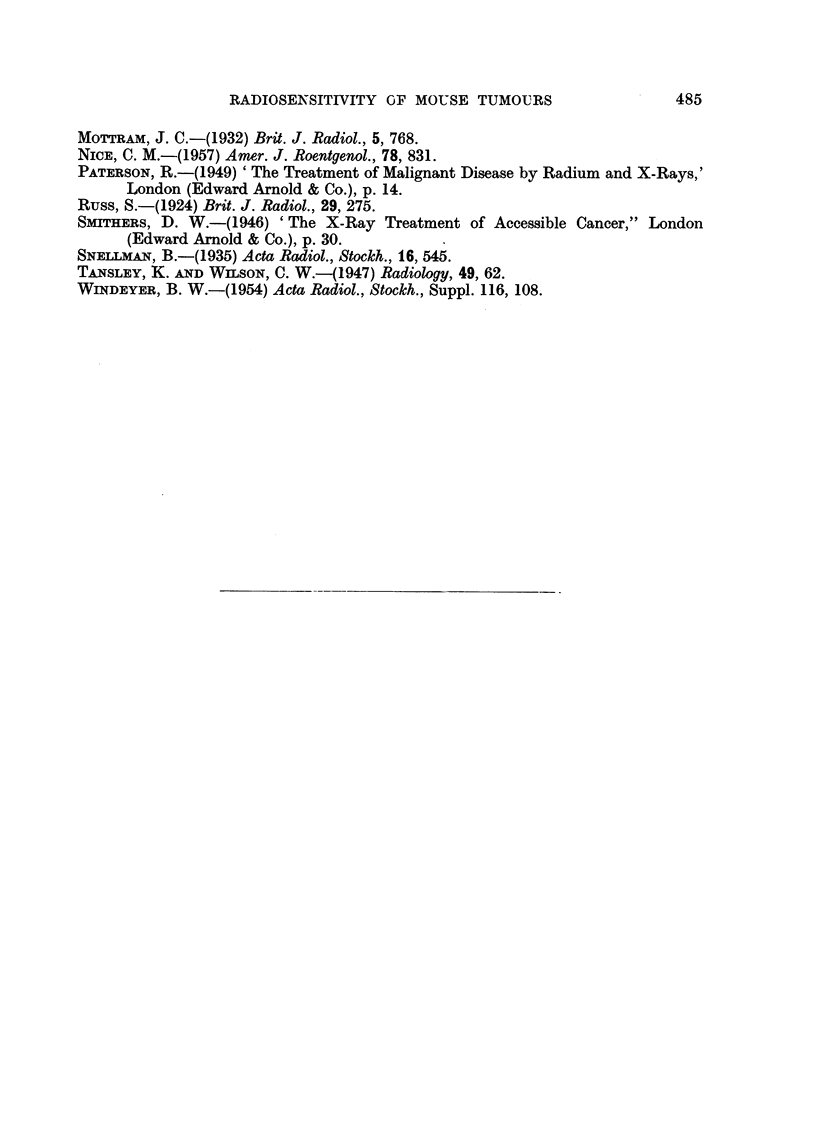# Serial Irradiation of Mouse Tumours: Changes in Radiosensitivity

**DOI:** 10.1038/bjc.1959.53

**Published:** 1959-09

**Authors:** A. E. G. Pearson


### SERIAL IRRADIATION OF MOUSE TUMOURS: CHANGES IN RADIOSENSITIVITY

A. E. G. PEARSON

*From the Department of Experimental Pathology, The Mount Vernon Hospital, Northwood, Middlesex*

Received for publication June 17, 1959

INCREASED radioresistance of a tumour after a number of sublethal irradiation doses have been administered, has been frequently reported clinically (Paterson, 1949; Cade, 1948; Windeyer, 1954). The action of the initial irradiation in selecting the more radioresistant cells from a heterologous population, inducing radioresistant mutants and the production of alterations in the tumour bed, have been proposed as explanations of acquired radioresistance (Conger and Luippold, 1957).

Experimental evidence of a direct selective or mutational effect on the tumour cells, resulting in a condition of increased radioresistance, has been contradictory. Irradiation lines of transplantable animal tumours, obtained by serial irradiations and transplantations into fresh animals, have been frequently employed to evaluate these changes. Hill, Morton and Witherbee (1919) employing mouse adenocarcinoma 63, Russ (1924), Mottram (1932) and Snellman (1935) on Jensen's rat sarcoma, and more recently Dittrich, Hohne and Schubert (1956) and Lettré (1956) on Ehrlich mouse carcinoma have claimed positive evidence of an increase in radioresistance. Negative evidence has been reported by Bagg (1938) on sarcoma 180, Montgomery and Warren (1953) and Nice (1957) on transplantable mouse lymphosarcomata, and Conger and Luippold (1957) and Baillif (1958) on Ehrlich mouse carcinoma.

The present study was undertaken to examine the effect on radiosensitivity of serial irradiations on transplantable mouse tumours, where the total administered dose reached a value many times the lethal level. The experiments cited above were conducted on well established tumours, usually of a non strain specific type. In this study two tumours arising in an inbred mouse strain were used as experimental material together with a homotransplant—sarcoma 37.

### MATERIALS AND METHODS

All experiments were conducted on RIII strain inbred mice. Sarcoma 37 was chosen for study owing to the regularity of its growth rate and absence of spontaneous regressions. The two homologous tumours were a spindle-celled sarcoma (BP1), induced in an inbred RIII strain mouse by subcutaneous injection of 3 : 4-benzpyrene colloid and passaged 6 times before the commencement of these experiments, and a spontaneous mammary adenocarcinoma (MV212) arising in the same mouse strain and previously passaged 17 times. All tumours were maintained as subcutaneous transplants by inoculation of 1 $mm.^3$ portions of actively growing tumour cortex into the right flank of recipient mice by means of a trocar and cannula. Sarcoma 37 and the spindle-celled sarcoma BP1 were maintained in male mice, and adenocarcinoma MV212 in females.

*Establishment of Serially Irradiated Tumour Lines*

Four irradiated tumour lines were established. Sarcoma 37, BP1 and MV212 received doses at each stage approaching the tumour lethal values (" B ", " F " and " G " lines, respectively), and sarcoma 37 irradiated with a half-lethal dose at each stage (" D " line). Subcutaneous tumours were irradiated when the external measurements approximated 15–30 mm.$^2$ in area (the product of the major and minor axes measured by calipers).

The doses administered at each stage in the irradiated series for the four tumour lines are given in Table I. 3000 r administered to sarcoma 37 produced tumour regression over a period of 15–20 days after treatment, usually to the point at which it ceased to become palpable. If growth resumed the tumour again became palpable within a few days. 2000 r administered to this tumour resulted in a slight tumour regression followed by growth cessation for a period of 12–20 days. The homologous tumours BP1 and MV212 reacted to sublethal doses in a less uniform manner; a cessation of growth, usually over a prolonged period, or greatly reduced growth rate followed a small initial regression. Tumours which became unpalpable did not recur. A proportion of tumours irradiated with doses well below the level required for all tumours to regress exhibited complete regression. For this reason the doses administered at each stage to these two homologous tumours were progressively reduced.

One tumour was selected at each stage, after recovery from irradiation, for transplantation into recipient mice by subcutaneous inoculation. Tumours developing in these mice were then irradiated and the series continued.

TABLE I.—*Irradiation Doses Administered to Each Stage of the Four Serially Irradiated Tumour Lines*

| Sarcoma 37 | | | | Sarcoma BP1 | | Adenocarcinoma MV212 | |
|---|---|---|---|---|---|---|---|
| " B " line | | " D " line | | " F " line | | " G " line | |
| B 0 | 3000r | D 0 | 2000r | F 0 | 3000r | G 0 | 2000r |
| B 1 | ,, | D 1 | ,, | F 1 | 2500r | G 1 | ,, |
| B 2 | ,, | D 2 | ,, | F 2 | 2000r | G 2 | 1500r |
| B 3 | ,, | D 3 | ,, | F 3 | 1750r | G 3 | 1250r |
| B 4 | ,, | D 4 | ,, | F 4 | 1500r | G 4 | ,, |
| B 5 | ,, | D 5 | ,, | F 5 | ,, | G 5 | ,, |
| B 6 | ,, | D 6 | ,, | F 6 | 1250r | G 6 | 1500r |
| B 7 | ,, | D 7 | ,, | F 7 | (from F 6 + 1250r) | G 7 | ,, |
| B 8 | ,, | D 8 | ,, | | | G 8 | ,, |
| B 9 | ,, | D 9 | ,, | | | G 9 | ,, |
| B 10 | ,, | D 10 | (from D 9 + 2000r) | | | G 10 | (from G 9 + 1500r) |
| B 11 | ,, | | | | | | |
| B 12 | ,, | | | | | | |
| B 13 | ,, | | | | | | |
| B 14 | ,, | | | | | | |
| B 15 | ,, | | | | | | |
| B 16 | 2500r | | | | | | |
| B 17 | (from B 16 + 2500r) | | | | | | |

Two sources of X-irradiation were used. For the establishment of the serially irradiated tumour lines a Siemens Stabilivolt Constant Potential Unit with a

Siemens EW 200 water-cooled glass tube were used. X-irradiation was generated at 190 kV and 8 mA. Filters were not employed and the dose rate in air at 25·5 cm. from the target was 240 r/min. having a quality of 0·25 mm. Cu H.V.L. Owing to a failure in this apparatus the radiosensitivity experiments and the irradiation of the last stages of the irradiated lines were conducted with X-ray apparatus employing a Siemens Doglas 200 kV tube maintained at the British Empire Cancer Campaign Research Unit in Radiobiology, at the Mount Vernon Hospital. Unfiltered radiation generated at 190 kV and 8 mA was again employed with this apparatus. The dose rate in air at 25·5 cm. from the target was 245 r/min. having a quality of 0·33 mm. Cu H.V.L.

Tumours were irradiated *in situ,* the mice being strapped to cork boards in the prone position ventral side down. The flap of the skin to which the tumour was adhering was lightly pulled away from the body and secured by adhesive plaster. This procedure was adopted to minimise the effect of scattered irradiation on the body. A lead shield with a suitable aperture over the tumour, protected the mouse.

External examination of the blood vessels in the skin flap, by means of a binocular microscope, showed that the degree of tension applied did not restrict the flow of blood.

*Radiosensitivity evaluations*

To assess the radiosensitivity of control and irradiated tumour lines, the proportion of " takes " of transplanted irradiated tumour fragments was taken as a criterion. A range of doses was employed up to the value where no tumours arose from the transplantation of the irradiated fragments ; this will be referred to hereafter as the lethal level.

Thin slices, not more than 1 mm. thick, from control and irradiated line tumours were placed on a No. 5 filter paper in a sterile 2-in. petri dish. The paper was marked to avoid confusion of the tumour slices. A medium of 60 per cent Earle's saline (Earle, 1943) 40 per cent horse serum was added, sufficient to cover the tumour slices. Each dish contained collateral control and treated tumour fragments and separate dishes were employed for each dose level. The dishes were irradiated at room temperature at a distance of 22 cm. from the target at 190 kV and 8 mA without filters ; the dose rate in air was 342 r/min. Subsequently the slices were cut into 1 mm. cubes and inoculated subcutaneously into the right flanks of 12 week old mice. Twelve mice were inoculated in control and treated groups for each dose level. These mice were kept for a period of two months after irradiation ; after this period animals showing no apparent external tumour were killed and the inoculation site examined. No apparently negative mice showed evidence of tumour establishment at autopsy.

In order to examine the possibility of the tumour bed modifying the effects of irradiation on control and treated tumours, *in vivo* irradiations with subsequent transplantation were carried out on sarcoma 37 and its serially irradiated " B " line. In these experiments four matched tumours from each group, from 50–70 sq. mm. in area, were irradiated at each dose level by the method described for the establishment of the irradiated lines. Immediately after irradiation the tumour cortex was excised to a depth of about 2 mm. from the region which faced the X-ray tube. This material was pooled within each group and placed in a glass homogeniser with 4 ml. of Earle's saline. Pulping was carried out until no perceptible fragments remained. This treatment had been found to give suspensions of single cells or clumps of not more than four cells. 0·1 ml. of the suspension was inoculated subcutaneously into the right flank of 30 mice in each group. These mice were examined over a period of two months for local tumour growth.

FIG. 1.—Radiosensitivity *in vitro* of sarcoma 37 and its serially irradiated lines " B " (sub-lethal dose at each stage) and " D " (half-lethal dose at each stage).

*In vitro* and *in vivo* radiosensitivity tests were conducted on sarcoma 37 and tumours from the 12th and 17th irradiation stages of the " B " line and only *in vitro* tests on the 10th stage of the " D " line. *In vitro* tests only were carried out on sarcoma BP1, the 7th irradiation stage of its " F " line, adenocarcinoma MV212 and the 10th stage of its irradiated " G " line. Owing to the restricted availability of the irradiation source and supply of mice, tumours from untreated sub-line passages from the specified irradiation stages had to be used for these tests.

### RESULTS

Fig. 1 illustrates the results from *in vitro* radiosensitivity tests on sarcoma 37, its derived " B " line tumours at the 12th and 17th irradiation stages (B 12 and B 17) and the 10th irradiation stage of the " D " line (D 10).

FIG. 2.—Radiosensitivity *in vivo* of sarcoma 37 and its serially irradiated " B " line.

B 12 tumours appeared to be more radiosensitive than the controls up to 2750 r. The lethal dose however was of the same order—3250 r (only one control tumour recurred at this dose level). B 17 tumours followed the same general pattern until the lethal dose was approached ; an apparent increase in radioresistance at 3250 r and 3500 r may be significant. Comparable results were obtained in the *in vivo* irradiation tests (Fig. 2) and B 12 tumours again exhibited an increase in radiosensitivity over the intermediate dose range of 1500–2500 r.

Increased radioresistance in " D " line tumours was observed over the intermediate dose range (Fig. 1) but again the lethal level was comparable with the controls.

The results of *in vitro* radiosensitivity tests on the two homologous tumours and their irradiated lines were comparable (Fig. 3 and 4). An increase in radioresistance was apparent in both irradiated lines with intermediate doses. At dose levels approaching the lethal values the response in control and treated groups was similar ; the lethal dose was the same for sarcoma BP1 and its irradiated " F " line and varied by only 250 r between adenocarcinoma MV212 and its irradiated " G " line.

FIG. 3.—Radiosensitivity *in vitro* of sarcoma BP1 and its serially irradiated " F " line.

### DISCUSSION

Two features emerge from these experimental findings. Firstly, the lethal dose levels of the irradiated tumour lines were not markedly different from their respective controls. Secondly, apparent differences in radiosensitivity were restricted to intermediate doses. Since the clinical observation of acquired radioresistance is related to complete tumour destruction, experimental comparisons should be made at this level. It would appear therefore from these experiments that the hypothesis of a selection by previous irradiations of more radioresistant cells, or a mutagenic effect, does not necessarily hold true. If the clinical evaluation of acquired radioresistance is based on experience of curability, with tumours of comparable position and histological pattern, a degree of uniformity in radiosensitivity would be presupposed. However, many authors have described wide variations in radiosensitivity among tumours of similar histological pattern, both in the clinical and experimental fields (Smithers, 1946 ; Cramer, 1934 ; Tansley and Wilson, 1947 ; Cohen and Cohen, 1955). Cathie (1939) from clinical experience did not regard histology as a good guide to sensitivity and a similar observation was made by Goldfeder (1947) on experimental material.

FIG. 4.—Radiosensitivity *in vitro* of adenocarcinoma MV212 and its serially irradiated " G " line.

The radiosensitivity comparisons between the control and irradiated lines indicate that a difference in response in the region of a tumour $LD_{50}$ dose cannot be extrapolated to provide information in the region of the lethal dose. The apparent increase in radioresistance in the irradiated lines of the two homologous tumours was observed only with intermediate doses. This effect may have been due to a synergistic effect of an independent factor, acting against the establishment of the tumour cells in the recipient host. The intensity of this factor may have been low, so that its action might be masked at the higher irradiation doses. This hypothetical factor may have been an immunity response by the host to the tumour implant. The previous irradiations might have modified the tumour cells with respect to their ability to produce antigens, or their reaction to the host antibodies. Cohen and Cohen (1953, 1954 *a*, *b* and *c*) have studied the relation between the host immunity response to a transplantable tumour and its radiosensitivity ; they considered that sensitivity was a function of the host resistance. If acquired radioresistance in clinical practice is a veridical phenomenon, it is suggested that immunological considerations may be involved in these isologous instances.

### SUMMARY

1. A method of establishing serially irradiated tumour lines of sarcoma 37 and two homologous tumours in an inbred mouse strain is described.

2. Radiosensitivity comparisons between the irradiated and control tumour lines have been made.

3. No marked differences in the lethal dose were observed between the four irradiated tumour lines and their respective controls.

4. Radiosensitivity differences were observed at intermediate dose levels. This was regularly consistent in the irradiated lines of the two homologous tumours and took the form of an increase in radioresistance.

5. The experimental results are discussed in relation to the problem of acquired radioresistance.

6. A possible relationship between these results and immunological factors is suggested.

My thanks are due to Dr. L. H. Gray, Director of The British Empire Cancer Campaign Research Unit in Radiobiology, The Mount Vernon Hospital, for irradiation facilities and to Mr. D. E. A. Jones and Dr. J. W. Boag for the dosimetry calculations. My thanks are also due to Mr. F. W. Butcher and Miss G. V. Adam for assistance with the animal experiments.

The expenses of this research were defrayed from a block grant by the British Empire Cancer Campaign.

### REFERENCES

BAGG, H. J.—(1938) *Amer. J. Roentgenol.*, **40**, 418.
BAILLIF, R. N.—(1958) *Abstr. Proc. Amer. Ass. Cancer Res.*, **2**, 277.
CADE, S.—(1948) ‘ Malignant Disease and its Treatment by Radium,’ 2nd Ed., London (John Wright & Sons). Vol. 1, p. 153.
CATHIE, A. B.—(1939) *Radiology*, **32**, 425.
COHEN, A. AND COHEN, L.—(1953) *Brit. J. Cancer*, **7**, 452.—(1954*a*) *Ibid.*, **8**, 303.—(1954*b*) *Ibid.*, **8**, 313.—(1954*c*) *Ibid.*, **8**, 522.—(1955) *Ibid.*, **9**, 600.
CONGER, A. D. AND LUIPPOLD, H. J.—(1957) *Cancer Res.*, **17**, 897.
CRAMER, W.—(1934) *Sci. Rep. Cancer Res. Fd., Lond.*, **11**, 127.
DITTRICH, W., HOHNE, G. AND SCHUBERT, G.—(1956) ‘ Progress in Radiobiology,’ Edinburgh (Oliver and Boyd) p. 381.
EARLE, W. R.—(1943) *J. nat. Cancer Inst.*, **4**, 165.
GOLDFEDER, A.—(1947) *Radiology*, **49**, 724.
HILL, E., MORTON, J. J. AND WITHERBEE, W. D.—(1919) *J. exp. Med.*, **29**, 89.
LETTRÉ, H.—(1956) *Ann. N.Y. Acad. Sci.*, **63**, 1022.
MONTGOMERY, F. O'B. AND WARREN, S.—(1953) *Radiology*, **60**, 421.

MOTTRAM, J. C.—(1932) *Brit. J. Radiol.*, **5**, 768.
NICE, C. M.—(1957) *Amer. J. Roentgenol.*, **78**, 831.
PATERSON, R.—(1949) 'The Treatment of Malignant Disease by Radium and X-Rays,' London (Edward Arnold & Co.), p. 14.
RUSS, S.—(1924) *Brit. J. Radiol.*, **29**, 275.
SMITHERS, D. W.—(1946) 'The X-Ray Treatment of Accessible Cancer," London (Edward Arnold & Co.), p. 30.
SNELLMAN, B.—(1935) *Acta Radiol., Stockh.*, **16**, 545.
TANSLEY, K. AND WILSON, C. W.—(1947) *Radiology*, **49**, 62.
WINDEYER, B. W.—(1954) *Acta Radiol., Stockh.*, Suppl. 116, 108.